# Can we trust measures of healthcare utilization from household surveys?

**DOI:** 10.1186/1471-2458-13-853

**Published:** 2013-09-17

**Authors:** Evelyn Korkor Ansah, Timothy Powell-Jackson

**Affiliations:** 1 Research & Development Division, Ghana Health Service, P.O. Box MB-190, Accra, Ghana; 2 Department of Global Health & Development, London School of Hygiene & Tropical Medicine, 15-17 Tavistock Place, London WC1H 9SH, UK

**Keywords:** Pictorial diary, Healthcare utilization, Recall periods, Health surveys

## Abstract

**Background:**

The research community relies heavily on measures of healthcare utilization from household surveys to understand health seeking choices and to evaluate interventions in developing countries. Such measures are known to suffer from recall problems but there is limited evidence of whether the method of data collection affects evaluation findings. We compared the results of a randomized trial of free healthcare using utilization data from two sources.

**Methods:**

Data are from a study in Ghana, in which 2,194 households containing 2,592 children under 5 y old were randomized into a prepayment scheme providing free primary and some referral care, or to a control group whose families paid user fees for healthcare. Data on morbidity and health seeking behaviour were collected using a standard household survey administered at endline and a pictorial diary given to households over a six month period, collected at monthly intervals.

**Results:**

Self-reported measures of morbidity and healthcare utilization were substantially lower in the household survey than the pictorial diary when the recall period was over a month. Introducing free healthcare had a positive effect on primary care visits based on the pictorial diary and a non-significant negative effect according to the household survey. Using any clinic visit in the past month as the outcome, the difference in the effect of free care between the two data collection methods was 3.6 percentage points (*p* = 0.078).

**Conclusions:**

The findings raise methodological concerns about measures of healthcare utilization from household surveys, particularly in the evaluation of health financing interventions.

## Background

Household surveys are frequently used to understand health seeking behaviour, particularly in developing countries where routine information systems may be unreliable for lack of investment and capacity. They are considered the standard tool for measuring healthcare utilization at the population level. A typical survey first asks household members whether they have been ill over a defined period and if so, whether they sought care and from whom. Recall periods vary between studies but standard practice is to extend the recall period to no more than one month in the case of outpatient care, and no more than a year in the case of inpatient care.

Measures of healthcare use from household surveys are widely used by the research community – to understand care seeking choices and to evaluate health programmes. Studies of the effect of health financing policies in developing countries rely heavily on such measures. Prominent examples in the recent literature include a randomized assessment of the Mexican universal health insurance programme [1] and various evaluations of the cooperative medical scheme in rural China [2,3]. Utilization measures from household surveys also provide the basis for conducting benefit incidence analyses in health [4,5].

To what extent can we trust measures of healthcare utilization from household surveys? There are several reasons to be cautious. First, individuals may struggle to recall episodes of illness and visits to healthcare providers. This failure to recall may vary systematically across population groups, thereby generating biases that have important implications for interpretation of the data. It is well documented, for example, that poorer individuals are less likely to report illness even though they experience greater morbidity [6]. A recent study in India experimentally assigned households to surveys with weekly and monthly recall periods, finding that the recall period had a large effect on reported morbidity, doctor visits and self-medication [7]. The impacts were particularly large for poorer households and those with a high burden of disease. However, when recall periods are shorter bias may be less of a problem. A study conducted in multiple developing countries showed that using a 7-day rather than a 2-day recall generated little bias in self-reports of illness [8]. Second, recall may vary according to the financing arrangements in place. If an individual has to pay out-of-pocket for care, he or she may be more likely to remember a doctor visit than someone who is covered by health insurance, particularly when it is costly. Such detection bias is why whenever feasible randomized controlled trials conceal assignment [9].

Much of the literature on problems with survey methods has addressed the issue of recall, but few studies have explored methods of data collection. In this paper we focus on the latter using data from rural Ghana. We first compare household survey measures of morbidity and healthcare utilization against our benchmark measure taken from a pictorial diary designed specifically for the study. We then take the novel step of comparing the results of a randomized trial of removing direct payments for healthcare using both sets of utilization measures. Our findings raise a number of concerns that have important methodological implications for our understanding of health reporting and the evaluation of policies on utilization of healthcare in resource-poor settings.

## Methods

### Study background

We used data collected for the purposes of a randomized experiment of removing direct payments for healthcare, in which one of the authors (EA) was the Principal Investigator. The study was conducted in Dangme West, a poor rural district in Southern Ghana with an estimated 2004 mid-year population of 115,000. The study provided free primary and some referral healthcare to households randomly assigned to the intervention group by paying for them to enrol into an existing prepayment health insurance scheme in May 2004. Households in the control group continued to pay a fee-for-service at public health facilities in accordance with the national policy at the time. Malaria was the leading cause of morbidity and mortality in children less than five years of age in the study district.

Households with at least one child aged 6 to 59 months and not already enrolled in the prepayment health insurance scheme were eligible to participate in the study. The sample frame consisted of approximately 8,700 households with children under five years of age living in the study area. Of the 2,332 households (with 2,757 children of eligible age) that were randomly selected from the district database to participate in the study, 138 households (with 165 children) had already enrolled voluntarily into the prepayment health insurance scheme by the time the registration window had closed. They were excluded from the main study but retained as an observational arm. The remaining 2,194 households with 2,592 children were randomly assigned to treatment and control groups.

At the baseline household survey in May 2004 a total of 2,151 households with 2,524 children were found and interviewed. In the final household survey, carried out at the end of the malaria transmission season in December 2004, 1,981 households with 2,321 children were successfully follow-up. In total, we have endline data for 2,319 children. Further details of the study – its design, the main outcomes and the findings – are available elsewhere [10,11].

### Measures of morbidity and healthcare utilization

We have two sources of data on healthcare utilization. The first was a panel household survey in which caretakers of the study children were interviewed before and after the introduction of the free care intervention. Questions relevant to this study included, “When was the last time your child fell ill?” and “When your child was last ill where did you seek care?” In contrast to most other household surveys, the questionnaire did not impose a specific recall period on the respondent. Rather, the first question was left open and responses were coded according to time categories. Based on responses to the two questions we construct binary indicators of the most recent illness, primary care clinic visit, and informal care visit for the following recall periods: past month, and past year. Informal care refers to traditional healers, chemists, or drug peddlars.

The second data collection method was a pictorial diary, supplied to households after the start of the intervention. This second source of data provides our benchmark or ‘gold standard’ measure of healthcare utilization. A diary is a research tool that requires respondents to make regular records of their daily activities and experiences. They are thought to be particularly useful in situations where there are likely to be difficulties in recalling past events, experiences or behaviours [12]. Diaries have been used as a data collection tool in many different fields of research. Some of these areas include nutrition [13], sexual behaviour [14-17], alcohol consumption [18,19] as well as lifestyle diaries [20]. Diaries are increasingly being mainstreamed into Living Standards Measurement Surveys conducted in numerous countries around the world as a means to capture information on household spending and consumption. The advantage of diaries is that the data are recorded as the events occur and therefore less likely to be affected by recall bias [21,22]. When used for a long period diaries can also provide a picture of variations in the behaviour under study. Conversely, the diary method may lead to a reduction in participant retention and compliance if the time and effort investment required is too high [21,23,24].

**Figure 1:**
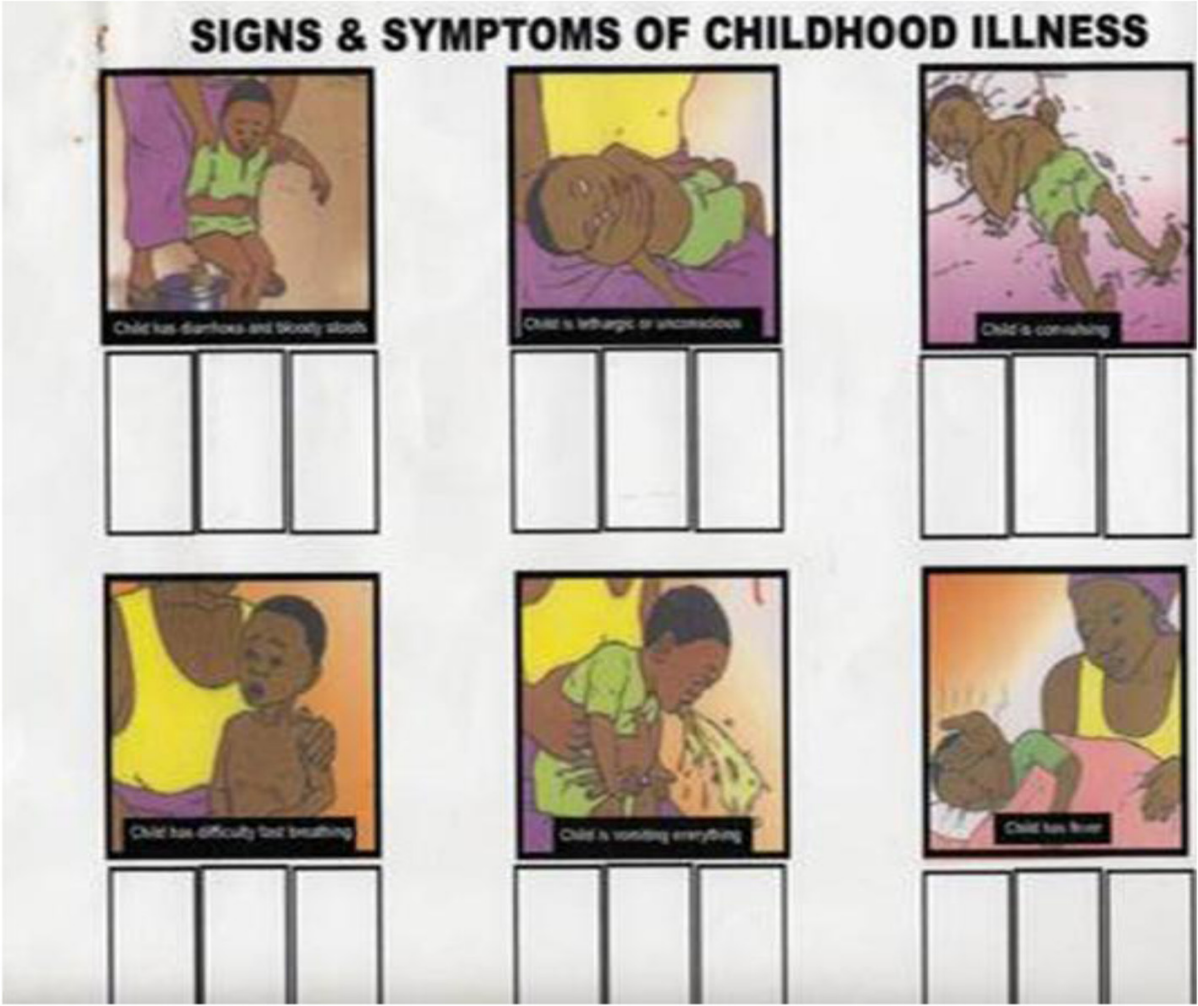
**Pictorial diary showing signs of childhood illness.** Picture 1 - Child has diarrhoea and bloody stools; Picture 2- Child is lethargic or unconscious. Picture 3 - Child is convulsing; Picture 4 - Child has difficulty in/fast breathing; Picture 5 - Child is vomiting everything; Picture 6 - Child has fever.

**Figure 2:**
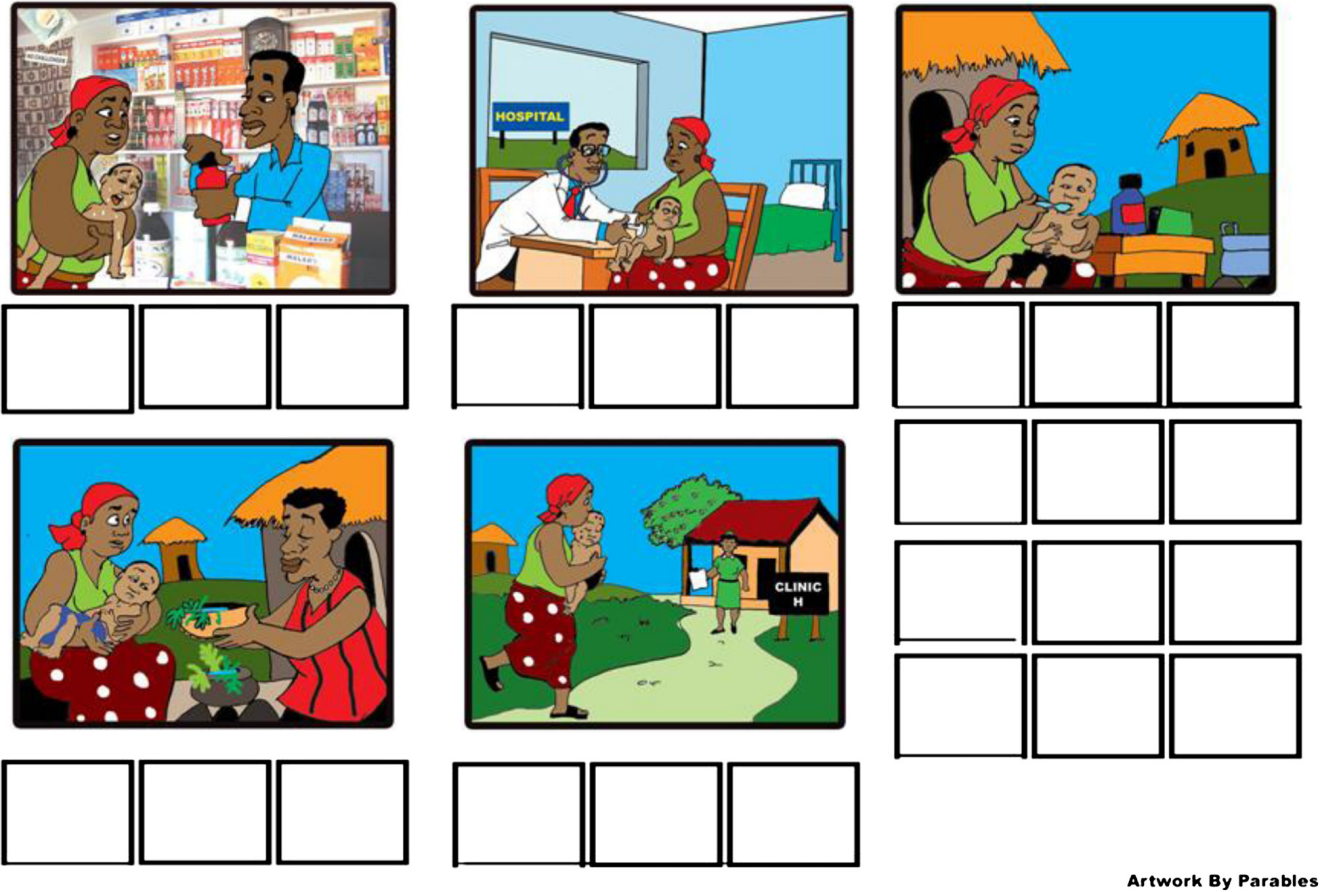
**Pictorial diary showing possible sources of healthcare.** Picture 1 - Chemical seller; Picture 2 - Hospital; Picture 3 - Treatment at Home; Picture 4 - Traditional Healer; Picture 5 - Primary Care Clinic.

A pictorial diary was developed specifically for this study to collect information from households on episodes of illness and healthcare seeking over a six month follow-up period (Figures 1 and 2). A key consideration to collecting reliable information was the fact that a substantial proportion of the mothers were illiterate. It was a two-part diary, the first of which depicted signs of common childhood illness (e.g. fever, vomiting, and convulsion). The pictures were adapted from the existing and well-known Road to Health Chart for children used widely in the country. The second part was designed by a local artist working closely with the Principal Investigator and depicted various healthcare seeking possibilities (e.g. primary care clinic, hospital, chemical seller, traditional healer and home). Both were thoroughly pre-tested locally for comprehension and feasibility.

The pictorial diaries were collected every month by trained fieldworkers. Caregivers were requested to make a mark underneath the relevant picture each time to indicate what illness the child suffered from and what healthcare (if any) was sought. Feedback from the study participants suggested that, anecdotally at least, the diaries were popular.

From the diary data we develop the following morbidity measures: annual number of illness episodes per person; any illness in the past month; and any illness over the six month diary data collection period. Corresponding measures are constructed for primary care clinic visits and informal care visits. For morbidity and utilization in the past month, we use data from the November pictorial diary to correspond with the month of recall in the household survey. In doing so we ensure that these measures are directly comparable between the two data sources, ruling out any influence of seasonality.

### Statistical analysis

There are several parts to the analysis. First, we compare mean values of morbidity and healthcare utilization from the two data sources, using the control group only. To assess the extent of under-reporting, we focus on measures of morbidity and utilization in the past month since these indicators are similarly defined. We also do the same for measures with the longer reference period (six months for the pictorial diary data and one year for the household data) while recognising that the comparison is not entirely valid.

Second, we estimate the effect of removing direct payments for healthcare on the use of primary healthcare and informal healthcare from the two sources of data. We use ordinary least squares and report both unadjusted estimates and estimates adjusted for age, education of mother, number of children in the household, household wealth, gender, distance to the nearest health facility, religion and ethnicity. In all regressions, we cluster the standard errors at the household level to deal with correlation between children in the same household and to account for the fact that treatment assignment was done on household basis.

Finally, we examine in a single regression framework whether the method of data collection influences the effect of the free care intervention on utilization of health services. We stack the utilization data from the two sources and run a regression in which we include a free care dummy, a household data collection dummy, and an interaction between the two. The coefficient on the interaction identifies the difference in the effect of free care between the two data sources. As before, we include controls and cluster the standard errors at the household level^a^.

## Results

Table 1 provides descriptive statistics of the morbidity and utilization measures. According to the pictorial diary data, 41% of children had at least one episode of illness in the past month, compared with 46% in the household survey data. When the reference period is extended, the difference between the two sources of data becomes large. The pictorial diary data show that 93% of children had at least one episode of illness over the six month period of data collection. By contrast, the proportion of children with any illness in the past year based on the household survey data was 60% despite the fact that the reference period is twice as long. These data suggest there was severe under-reporting of illness in the household survey. At least one third of morbidity (33%) – and probably more if the reference periods were identical – was not captured by the household survey.

**Table 1:** Summary statistics on morbidity and healthcare utilization from the pictorial diary and household survey data

	**Mean**	**Standard deviation**	**N obs**
	**(1)**	**(2)**	**(3)**
	Panel A: Pictorial diary measures		
*Morbidity*			
Any illness past one month	0.410	0.410	1,197
Any illness past six months	0.928	0.258	1,197
Illness episodes per year	9.77	8.11	1,197
*Healthcare utilization*			
Any clinic visit past one month	0.180	0.384	1,197
Any clinic visit past six months	0.629	0.483	1,197
Clinic visits per year	2.52	2.81	1,197
Any informal care visit past one month	0.157	0.364	1,197
Any informal care visit past six months	0.670	0.470	1,197
Informal care visits per year	3.08	3.35	1,197
	Panel B: Household survey measures		
*Morbidity*			
Any illness past one month	0.457	0.498	1,193
Any illness past one year	0.604	0.489	1,193
*Healthcare utilization*			
Any clinic visit past one month	0.153	0.360	1,193
Any clinic visit past one year	0.217	0.412	1,193
Any informal care visit past one month	0.211	0.408	1,193
Any informal care visit past one year	0.267	0.443	1,193

Notes: Data are for the control group only. The number of illness episodes and clinic visits per year from the pictorial diary are based on data collected over a six month period. To account for seasonality, any illness, clinic visit, or informal care visit in the past month based on the pictorial diary is for the month of November so as to be comparable with the month of recall in the household survey.

The proportion of children who sought healthcare in the past month is reasonably similar across the two sources of data in the case of both clinic visits (18% pictorial diary vs. 15% household survey) and informal care visits (16% pictorial diary vs. 21% household survey). When the reference period is lengthened to six months/ one year, the method of data collection appears important in the measurement of any clinic visit (63% pictorial diary vs. 22% household survey) and informal care visits (67% pictorial diary vs. 27% household survey).

Table 2 reports the effect of removing direct payments for healthcare across the different measures of utilization. The intervention had a positive effect on primary care visits according to the pictorial diary data (Table 2, Panel A). The unadjusted estimates indicate it increased the number of visits by 0.30 per year (unadjusted rate ratio of 12 percent) and the probability of any visit in the past six months by 3.7 percentage points (unadjusted rate ratio of 6 percent). According to the household survey data, the intervention had no statistically significant effect on clinic use (Table 2, Panel B). The point estimates are negative suggesting that, if anything, the intervention reduced primary care use. The point estimates for informal care utilization are negative irrespective of which data collection method was used. Only in the case of informal care visits from the pictorial diary is the estimate significant, with a negative effect of 0.27 per year (unadjusted rate ratio of 9 percent).

**Table 2:** The effect of free care on healthcare utilization from the pictorial diary and household survey data

	**Control group**	**Unadjusted effect**	**Adjusted effect**	**95% CI**	**p-value**
	**(1)**	**(2)**	**(3)**	**(4)**	**(5)**
	Panel A: Pictorial diary method of data collection				
Any clinic visit past one month	0.180	0.014	0.015	−0.019, 0.048	0.387
Any clinic visit past six months	0.629	0.037	0.035	−0.006, 0.075	0.094
Clinic visits per year	2.518	0.298	0.286	0.037, 0.534	0.024
Any informal care visit past one month	0.157	−0.011	−0.012	−0.043, 0.019	0.453
Any informal care visit past six months	0.670	−0.021	−0.023	−0.063, 0.017	0.257
Informal care visits per year	3.081	−0.270	−0.290	−0.568, -0.012	0.041
	Panel B: Household survey method of data collection				
Any clinic visit past one month	0.153	−0.022	−0.023	−0.052-0.006	0.122
Any clinic visit past year	0.217	−0.017	−0.017	−0.051-0.016	0.315
Any informal care visit past one month	0.211	−0.011	−0.013	−0.048, 0.022	0.470
Any informal care visit past year	0.267	−0.023	−0.023	−0.060, 0.014	0.229

Notes: Adjusted estimates include controls for mother’s education, number of children in household, age of the child, household wealth and dummies for male child, distance from the nearest health centre, religion and ethnicity. The 95% CI and p values are based on standard errors that are corrected for clustering at the household level.

Table 3 shows the extent to which the method of data collection affects the estimated impact of the free care intervention on healthcare utilization by reporting the coefficient on the interaction between free care and the data collection method. With respect to clinic visits, the data collection method appears to matter. Depending on the reference period, the effect of free care on use of clinic visits is 3.6 percentage points to 5.4 percentage points smaller with the household data. In the case of informal care visits, there are no differences in the effect of free care with respect to the data collection method.

**Table 3:** The effect of method of data collection on healthcare utilization findings of free care experiment

	**Coefficient on interaction**	**95% CI**	**p-value**	**N**	**R**^**2**^
	**(1)**	**(2)**	**(3)**	**(4)**	**(5)**
Any clinic visit past one month	−0.036	−0.076, 0.004	0.078	4,632	0.0118
Any clinic visit past six months/one year	−0.054	−0.101, -0.007	0.024	4,632	0.2110
Any informal care visit past one month	−0.00037	−0.041, 0.040	0.986	4,632	0.0150
Any informal care visit past six months/ one year	−0.0017	−0.051, 0.048	0.948	4,632	0.1805

Notes: Data are stacked such that each observation is a child corresponding to one of the two data collection methods. The coefficient reported is on the interaction between a dummy for free healthcare and a dummy for whether the data were collected through the household survey. It identifies the difference in treatment effect between the pictorial diary and household data. The 95% CI and p values are based on standard errors that are corrected for clustering at the household. All regressions control for mother’s education, number of children in household, age of the child, household wealth and dummies for male child, distance from the nearest health centre, religion, ethnicity, free healthcare and the method of data collection.

## Discussion

Using pictorial diary data as our benchmark, we addressed the question of whether household survey data on health seeking behaviour provide reliable measures of healthcare use. We found substantial under-reporting of morbidity and healthcare utilization when recall periods were extended beyond one month and qualitatively different conclusions as to the effect of removing user fees on primary care use. On a practical level the study demonstrated the feasibility of implementing pictorial diaries in a rural population where a sizeable proportion of respondents were illiterate. The caregivers did not appear to have any difficulty in using the pictorial diaries as a means of reporting health events.

The severe under-reporting of morbidity was consistent with a study in India examining how different recall periods affect reporting [7]. Since morbidity acts as the standard screening question prior to asking about health seeking behaviour in household surveys, this result inevitably implies substantial under-reporting of healthcare use. The findings were also consistent with those of a study in the US on alcohol consumption and sexual activity which found that participants more often underreported their sexual activities and condom use during retrospective studies as compared to when diaries were used [17].

Evidence on how the survey method alters the conclusions of the randomized trial of removing direct payments for healthcare is the most novel aspect of the paper. The change in the direction of effect between data sources is both intriguing and worrisome, given the ubiquitous use of household survey data in the evaluation of health policies. We may have viewed the findings with more scepticism had they been based on an observational study. But the randomized design and the fact that the treatment effects are negative for both of the two household survey measures of primary care utilization lend credibility to the findings.

We can only speculate as to the reason for this finding but one hypothesis is that the recall of families in the intervention group deteriorated precisely because they no longer had to pay for healthcare, making the event less salient and more easily forgotten, thereby dampening the true effect of the intervention^b^. If so it would provide an example of how detection bias can emerge passively through the influence of an intervention on the recall of self-reported behaviours. Because the intervention did not affect the price of care at informal providers the salience and recollection of informal care seeking events should not have been affected by the intervention. Indeed our findings regarding the effect of the intervention on informal care visits were consistent across the two methods of data collection, providing further support to this hypothesis.

The study had a number of limitations that should be noted. First, although the contribution of the study is methodological, it is based on empirical findings from one district in Ghana. However, there is good reason to believe recall problems are universal. Extensive evidence from the US showing that around 20 percent of outpatient events are under-reported when the recall period extends beyond two to three days [25] has been confirmed by studies in developing countries [26,27]. Second, owing to data limitations we were only able to construct one indicator of utilization that was standardised across the two data collection methods. A greater number of comparable measures of utilization would have strengthened the analysis. Finally, our method does not allow recall effects to be separated from data collection methods, although it seems likely the channel through which the data collection method altered the effect of free care is via recall.

## Conclusions

The findings raise methodological concerns about measures of healthcare utilization from household surveys, particularly in the evaluation of health financing interventions. Further research is needed into the problems of bias when collecting utilization data in household surveys. Such data are widely used by health researchers to understand health seeking behaviour, in benefit incidence analysis and in the evaluation of interventions and policies. The implications of the study’s findings are therefore potentially far reaching. It may be the case that the trend towards using household diaries of income and expenditure should be extended to the collection of data on healthcare utilization.

### Endnotes

^a^We ran regressions in which we included child fixed-effects instead of the demographic controls. The results are very similar to those produced in Table 3. The findings are also qualitatively the same if we use a logit model instead of OLS.

^b^There is a suggestion in the paper by Das et al. (2012) that much of what is underreported in household surveys when the recall period is relatively long are the less severe illnesses associated with smaller household expenditures [7].

## Abbreviations

US: United States.

## Competing interests

The authors declare that they have no competing interests.

## Authors’ contributions

EA designed the evaluation, carried out the data collection, contributed to the analysis and drafting of the manuscript. TPJ carried out the analysis and drafting of the manuscript. Both authors read and approved the final manuscript.

## Pre-publication history

The pre-publication history for this paper can be accessed here:

http://www.biomedcentral.com/1471-2458/13/853/prepub
